# Development of an Electronic Portal Imaging Device Dosimetry Method

**DOI:** 10.3390/diagnostics11091654

**Published:** 2021-09-09

**Authors:** Jun Zhang, Ziting Fan, Xile Zhang, Ruijie Yang, Junhai Wen

**Affiliations:** 1 Department of Biomedical Engineering, School of Life Science, Beijing Institute of Technology, Beijing 100081, China; 3120160563@bit.edu.cn (J.Z.); fdlhlh@163.com (Z.F.); 2 Department of Radiation Oncology, Peking University Third Hospital, Beijing 100191, China; xlz9568@163.com (X.Z.); ruijyang@yahoo.com (R.Y.)

**Keywords:** radiotherapy, quality assurance, EPID, portal dosimetry, backscatter, off-axis

## Abstract

Support arm backscatter and off-axis effects of an electronic portal imaging device (EPID) are challenging for radiotherapy quality assurance. Aiming at the issue, we proposed a simple yet effective method with correction matrices to rectify backscatter and off-axis responses for EPID images. First, we measured the square fields with ionization chamber array (ICA) and EPID simultaneously. Second, we calculated the dose-to-pixel value ratio and used it as the correction matrix of the corresponding field. Third, the correction value of the large field was replaced with that of the same point in the small field to generate a correction matrix suitable for different EPID images. Finally, we rectified the EPID image with the correction matrix, and then the processed EPID images were converted into the absolute dose. The calculated dose was compared with the measured dose via ICA. The gamma pass rates of 3%/3 mm and 2%/2 mm (5% threshold) were 99.6% ± 0.94% and 95.48% ± 1.03%, and the average gamma values were 0.28 ± 0.04 and 0.42 ± 0.05, respectively. Experimental results verified our method accurately corrected EPID images and converted pixel values into absolute dose values such that EPID was an efficient radiotherapy dosimetry tool.

## 1. Introduction

Intensity-modulated radiation therapy (IMRT) and volumetric-modulated arc therapy (VMAT) are widely used in radiotherapy because they can ensure that the tumor area receives higher and more conformal doses and reduce unnecessary radiation exposure of the surrounding normal tissues. Because of its design and implementation complexity, patient-specific dose verification has been recommended to guarantee the delivery dose is consistent with the prescribed dose [1].

An electronic portal imaging device (EPID) was originally used for patient setup verification. Due to its large imaging area, fast acquisition speed, high resolution, good linear response, long-term measurement stability, and being mounted on the linac, EPID has been extensively used in IMRT and VMAT quality assurance [2,3,4,5,6,7], including pretreatment and in-vivo dose verification. Pretreatment dose verification usually involves the following two approaches: first, the fluence generated by the treatment planning system (TPS) is convolved with the kernel to predict the EPID image; then, the predicted image is compared with the measured EPID image for verification [8,9,10]. This method is widely used in clinics, but may not reflect the errors introduced in the TPS calculation process. Second, the EPID image is converted into a dose value and compared with an independent verification system [6,11,12,13]. Many studies have shown that EPID has the potential to be used as a direct dosimetry tool [14,15,16,17,18] and to detect patient related errors during radiotherapy [19,20]. When some of Varian’s EPID are exploited for dose measurement, two factors may affect the dosimetric properties of EPID. One factor is that EPID is mounted on the linac by means of a support arm, and the low-energy scatter ray produced by the interaction between X-rays and the support arm may lead to asymmetry of the EPID image in the inline direction [13,21,22,23,24,25]; relevant studies show that the effect may exceed 5% [26,27]. The other factor is that EPID requires dark field and flood field calibration to ensure that the response of each pixel is constant, which results in the off-axis response of EPID being different from the actual output of the linac [28,29]. Therefore, when EPID is used as a dosimetry tool, these two factors must be corrected before converting pixel values into dose values.

Several methods to correct the backscatter of EPID exist. First, Monte Carlo (MC) simulation or measurement data are used to model the backscatter kernel [30]; then, the deconvolution method is used to eliminate the influence of the support arm. The drawback of this method is that the MC approach requires detailed physical modeling of EPID, and the modeling process is complex. Moreover, to increase the calculation speed, convolution or deconvolution is generally applied in the frequency domain via fast Fourier transform [25,31]; in this situation, the kernel is required to have no spatial deformation; that is, each pixel of EPID applies the same backscatter kernel, which is not consistent with the actual situation. Second, a lead plate is placed under the EPID or above the support arm to create a shielding layer, and the influence of backscatter is calculated by comparing the off-support arm image [22,24,26,27]. The limitation of this method is that the lead increases the weight of EPID and introduces a uniform scatter kernel, which changes the dosimetric properties of EPID. In addition, the disassembly and installation of the support arm require a professional, and the process is time-consuming. Third, the linear correction functions corresponding to different fields are calculated by comparing the value in the inline positive direction with the negative direction or the crossline direction [21,25]. However, since different EPIDs have different responses, this linear correction relationship does not apply to all EPIDs.

For the off-axis correction of EPID, Greer [26] and Rowshanfarzad et al. [22] derived a pixel sensitivity matrix from EPID images with and without a support arm to replace the flood field. This method required disassembly of the support arm, and the process was cumbersome. Camilleri et al. [25] obtained the correction matrix as the ratio of the EPID image and the dose value in water scanned by the ionization chamber, but the process of measuring the dose using a water tank is time-consuming.

The ionization chamber array (ICA) can accurately measure the output dose of the linac, but it cannot rotate with the gantry, and its application in the treatment process is a challenge. The EPID is convenient to use, but it cannot directly measure the output dose of the linac. In our study, we developed a novel method to accurately correct the EPID backscatter and off-axis response, so that EPID can replace the ICA to measure the output of the linac and can be utilized to detect errors in radiotherapy. Taking the measured values of the ICA as a reference, we established the correction matrix for different EPID images according to the EPID response. The matrix is suitable for both EPID backscatter and off-axis response correction. The corrected EPID image was converted to absolute dose values by means of the pixel value and dose conversion function. Our method is simple to implement compared with other methods, and it does not require separate calculation of the backscatter kernel for different fields. The backscatter and off-axis responses of different fields can be corrected using the same matrix.

## 2. Materials and Methods

### 2.1. Materials

All measurements were performed on a Trilogy linac (Varian Medical Systems, Palo Alto, CA, USA) using 6 MV X-rays. The linac is equipped with an amorphous silicon EPID (aS1000), covering a field size of 40 × 30 cm^2^ with a resolution of 1024 × 768 pixels. EPID images were captured using the integrated model, and the acquisition software was IAS3 (Image Acquisition System 3). Dark and flood fields were acquired before the experiment to correct raw images using Formula (1). The ICA used for dose measurement was MatriXX (IBA Dosimetry, Schwarzenbruck, Germany), which consists of 1020 ion chambers arranged in a 32 × 32 grid covering an effective detection area of 24 × 24 cm^2^. The diameter of the ionization chamber is 0.45 cm, and the center-to-center distance is 0.76 cm. The MatriXX has a build-up layer of 0.3 cm, and 1.2 cm of solid water was placed above it as an additional build-up layer and 5 cm backscatter solid water was placed below it. Both EPID and MatriXX were mounted on the isocenter (SDD = 100 cm), and the gantry angle was set to 0 degrees.

(1) $$G_{\mathrm{EPID}}=\frac{G_{\mathrm{raw}}-DF}{FF-DF}\cdot FF_{\mathrm{mean}}$$

where $G_{\mathrm{raw}}$ is the raw image acquired by EPID, DF is the dark field, *FF* is the flood field, $FF_{\mathrm{mean}}$ is the average value of the flood field, and *G_EPID_* is the modified EPID image. 

### 2.2. Methods

The purpose of this work was to convert EPID images into absolute dose values. The procedures include backscatter correction, off-axis response correction, field output correction, and absolute dose correction, as shown in Formula (2).

(2) $$D_{\mathrm{EPID}}=f\{G_{\mathrm{EPID}}\cdot CM\cdot OF\}$$

where $D_{\mathrm{EPID}}$ is the absolute dose value calculated by the EPID pixel value, f is the conversion function between the pixel and dose values, $G_{\mathrm{EPID}}$ is the pixel value of the EPID image, CM is the backscatter and off-axis correction matrix, and OF is the field output correction factor.

#### 2.2.1. EPID Image Preprocessing

When performing backscatter and off-axis response correction on EPID images, the measured dose of ICA was taken as the reference value. Since the diameter of the ionization chamber in MatriXX is 0.45 cm and the pixel size of EPID is 0.039 cm, the average value of 12 × 12 pixels around each EPID pixel was exported to match the measured area of the ionization chamber during the modeling process.

#### 2.2.2. Backscatter and Off-Axis Response

**Figure 1:**
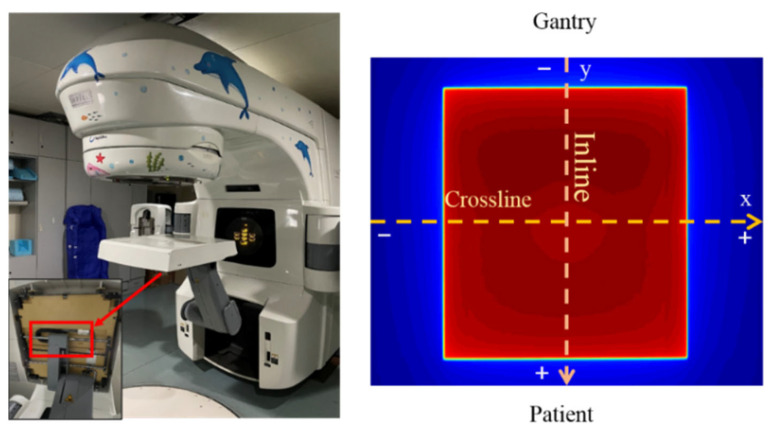
The structure of the EPID support arm (**left**) and the acquired EPID image (**right**).

The EPID is mounted on the linac and rotated with the gantry. X-rays passing through the EPID and interacting with the support arm generate scattered X-rays, resulting in asymmetry in the inline direction of the EPID image (Figure 1). In addition, the beam profile “horn” is removed after the dark and flood field calibration. Therefore, when EPID is used for dosimetry, the effects of backscatter and off-axis response must be corrected. The ICA has been universally applied in dose measurement in radiotherapy. It is not affected by backscatter and off-axis responses and can accurately measure the output characteristics of the linac. Consequently, the backscatter and off-axis response of EPID can be corrected by means of the measured value of the ICA. The detailed procedure is as follows.

**Figure 2:**
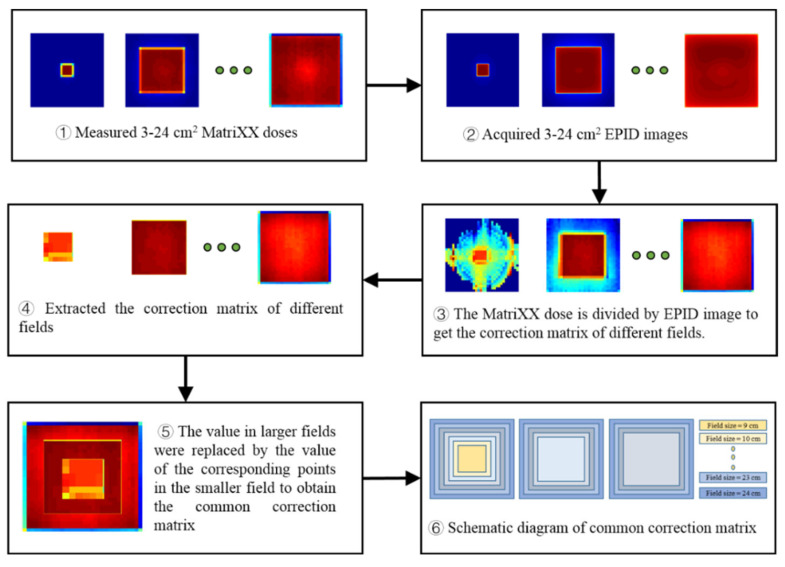
The generation process of the common correction matrix.

First, the pixel and dose values corresponding to square fields of 3–24 cm^2^ with 100 MU were measured by EPID and ICA and then normalized to the central axis. The pixel value of EPID corresponding to the measurement point of ICA was extracted, and the ratio of the dose value to the extracted pixel value was taken as the correction matrix of different fields. Second, the correction matrix value within the penumbra region (20% of the maximum dose) of different fields was extracted, and the values in larger fields were replaced by the values of the corresponding points in the smaller fields to obtain the common correction matrix. The generation process of the common correction matrix is shown in Figure 2. Due to the different influences of backscatter on different EPID images, we use correction matrices of 9–24, 13–24 and 18–24 cm^2^ fields to produce three common correction matrices suitable for different fields. These three matrices can be used to correct the backscatter and off-axis response of EPID images for different fields.

#### 2.2.3. Conversion of EPID Pixel Value and Dose Value

There is a linear relationship between the EPID pixel value and dose value [32]. The EPID pixel and ICA dose values were acquired after irradiation with 1–600 MU over a 24 × 24 cm^2^ field at the maximum dose rate (600 MU/min). The dose and EPID pixel values corrected by the backscatter and off-axis response corresponding to different off-axis points were extracted, and the function used to convert the pixel value into the dose value for different off-axis points was applied:

(3) $$D_{\mathrm{EPID}}(r)=a(r)\cdot G_{\mathrm{EPID}}(r)+b(r)$$

where r ($r=\sqrt{x^{2}+y^{2}}$, x and y are the coordinate index of each point in the EPID plane) is the off-axis distance, a and b are dose conversion coefficients.

#### 2.2.4. Field Output Factor

For the same field, the scattered rays cause a different response of EPID and ICA, which leads to different pixel and dose conversion functions corresponding to different fields. To use the uniform conversion function for different fields, we modify it using the field output factor. The pixel value of EPID and the absolute dose of ICA at the central axis were acquired for 3–24 cm^2^ fields and 100 MU. All of the values were normalized to the measured value of the 24 cm^2^ field. The field output factor was derived from the ratio of the pixel value and absolute dose.

### 2.3. Validation

A total of 103 fields corresponding to 30 IMRT plans were acquired for verification. All measurements were performed with 6 MV X-ray beams and delivered using the sliding window technique. The EPID images were corrected by the correction matrix and the field output factor and converted to absolute dose values. Under the same conditions, MatriXX was used to measure the absolute dose values. The calculated and measured values were compared using global gamma analysis of 3%/3 mm and 2%/2 mm (5% threshold) to verify the model’s accuracy.

## 3. Results

### 3.1. Backscatter and Off-Axis Response Correction

**Figure 3:**
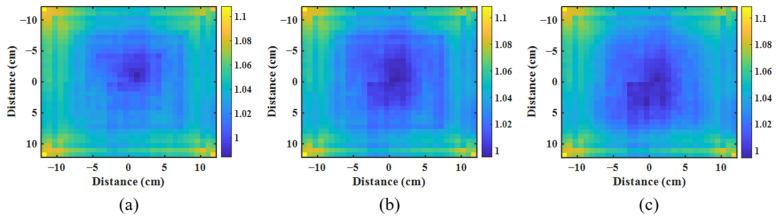
EPID correction matrices of backscatter and off-axis response. (**a**–**c**) are correction matrices composed of 9–24 cm^2^, 13–24 cm^2^ and 18–24 cm^2^ fields respectively.

**Figure 4:**
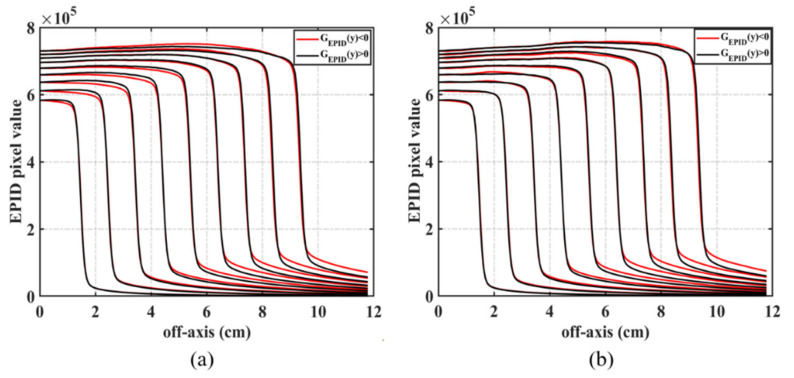
Values of the uncorrected (**a**) and corrected (**b**) EPID images in the inline direction for different fields.

Different EPID images have different effects on backscatter. We acquired the EPID image and ICA dose value of different field sizes and calculated the backscatter correction matrix corresponding to each square field. Then, three common correction matrices of backscatter and off-axis responses were generated (Figure 3). Figure 4a shows the symmetry of the EPID image in the inline direction. When the field size is ≤10 cm^2^, the value in the positive direction is greater than that in the negative direction; when 10 cm^2^ < field size ≤ 15 cm^2^, the value symmetry is good; and when the field size is >15 cm^2^, the value in the positive direction is smaller than that in the negative direction. In these three cases, the correction of backscatter used in the correction matrix was composed of 9–24 cm^2^ (Figure 3a), 13–24 cm^2^ (Figure 3b), and 18–24 cm^2^ (Figure 3c) fields. After correction, the pixel value of EPID had good symmetry in the inline direction (Figure 4b).

**Figure 5:**
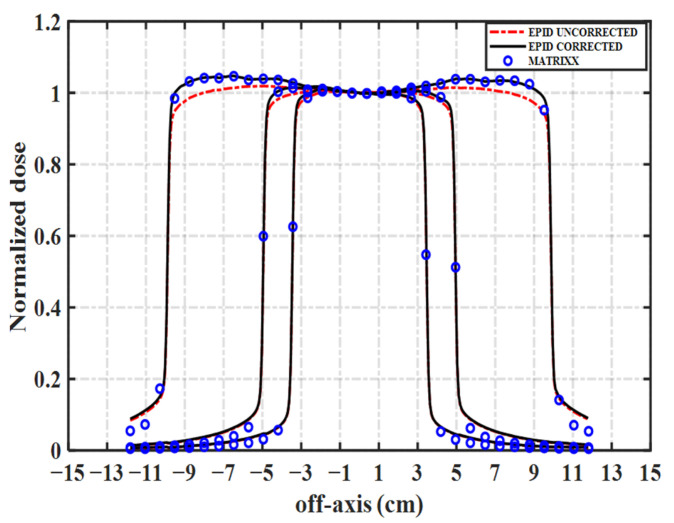
Comparison of uncorrected and corrected EPID images with the measured value of ICA in the inline direction.

We compared the EPID images with the dose values measured by the ICA to illustrate the variation in the EPID off-axis response. Figure 5 compares uncorrected and corrected EPID images and the corresponding measurement values of ICA in the inline direction when the field size is 7, 12, and 23 cm^2^, where all of the values were normalized to their respective central axis. As the off-axis distance increases, the deviation between the uncorrected EPID image and the measured value of ICA gradually increases. The off-axis response of the corrected EPID image is consistent with the measured value of ICA.

### 3.2. Pixel Value and Dose Conversion Function

We used EPID and ICA to measure the pixel and dose values for 1–600 MU when the field was 24 cm^2^. The measured value of each off-axis point was extracted to calculate the conversion function between the pixel and dose values.

**Figure 6:**
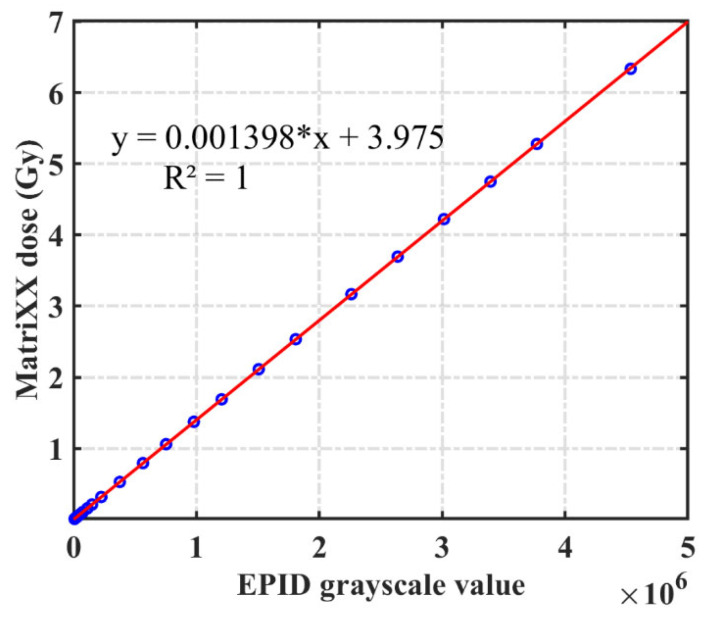
Pixel value and dose conversion function of the central axis in 24 cm^2^ field.

Figure 6 shows the linear relationship between the pixel and measurement dose values of ICA at the central axis, the measurements were repeated three times and their averages were used to calculated a(r) and b(r). The model described those factors with a maximum error of 0.42% between measured and calculated values. Table 1 depicts the corresponding coefficients of the function of each off-axis point in the corrected EPID image and uncorrected EPID image. The coefficients corresponding to different off-axis points in the uncorrected EPID images increase as the off-axis distance increases. After correction, the coefficients of the different off-axis points remain consistent.

Table 2 shows the error between the measured dose of the ICA and the calculated dose of EPID. The different off-axis points of the corrected and uncorrected EPID use the central axis coefficient to calculate the dose value. The error between the calculated dose value of the corrected EPID image and the measured dose value is less than 0.5%. For the uncorrected EPID image, the error increases gradually with increasing off-axis distance, and the relative error is 5% when the off-axis distance is 11 cm. Therefore, the central axis conversion coefficient can be used to calculate the dose value of each point in the corrected EPID image.

### 3.3. Field Output Factor

**Figure 7:**
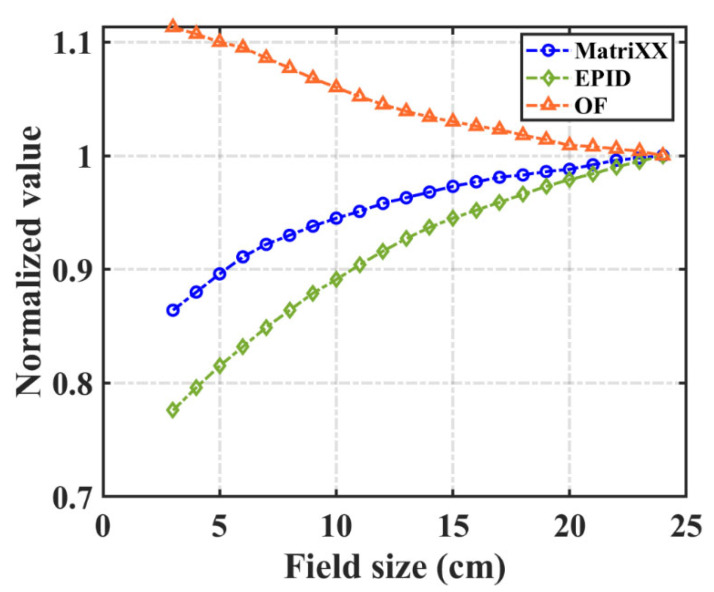
The EPID pixel value, MatriXX dose value and field output factor (OF) at the central axis of different fields. All values were normalized to the field of 24 × 24 cm^2^.

In Figure 7, the EPID pixel value, dose value measured by ICA and corresponding field output factor at the central axis of different fields are normalized to the field value of 24 × 24 cm^2^. With increasing field size, the pixel value of EPID and the dose value of ICA both increase, and the field output correction factor decreases.

### 3.4. Dose Comparison for IMRT Fields

A total of 103 IMRT fields were acquired for verification. Both EPID and ICA were placed at 100 cm. The corrected EPID pixel value was converted into the absolute dose value and compared with the value measured by ICA using the global gamma analysis of 3%/3 mm and 2%/2 mm pass criteria, with a dose threshold of 5%. The gamma pass rates were 99.6% ± 0.94% and 95.48% ±1.03%, and the mean gamma values were 0.28 ± 0.04 and 0.42 ± 0.05, respectively.

**Figure 8:**
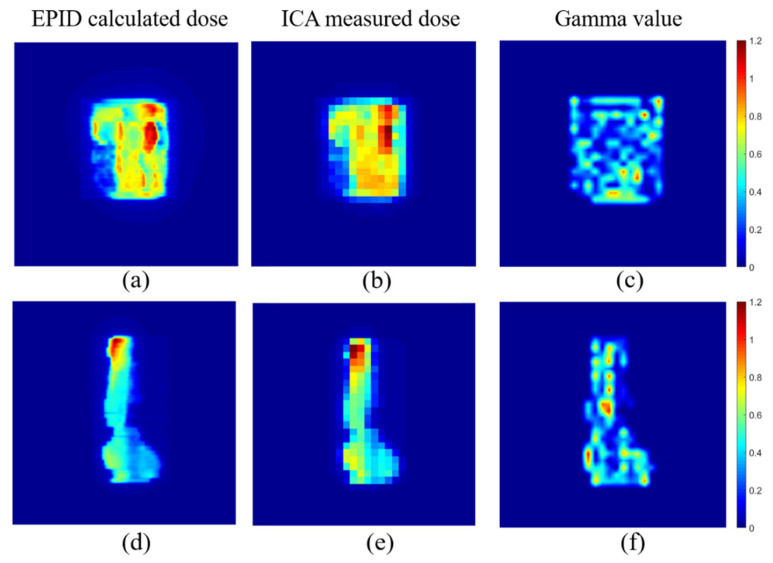
EPID calculated dose value (**a**,**d**), ICA measured dose value (**b**,**e**) and corresponding 2%/2 mm gamma value (**c**,**f**).

Figure 8 compares the dose value converted from the EPID pixel value and the dose measured by ICA of two IMRT fields.

## 4. Discussion

**Figure 9:**
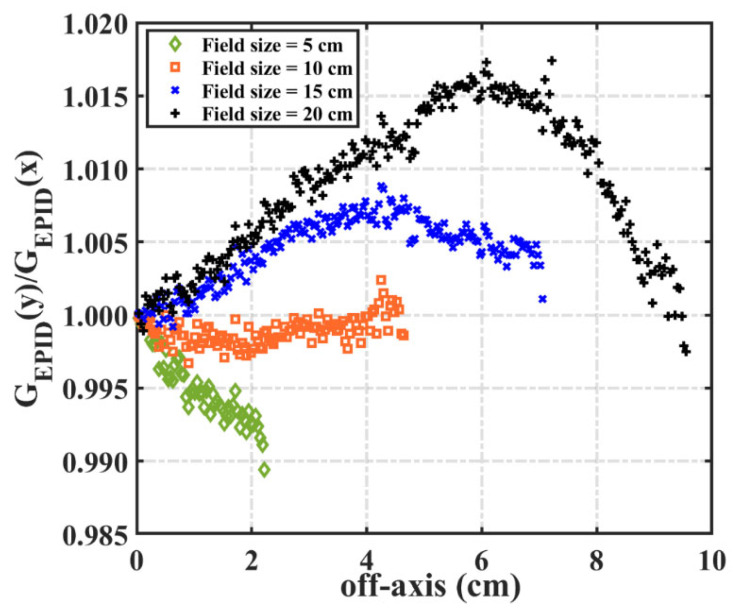
The ratio of the EPID pixel values in the negative inline and crossline directions.

We developed a novel method to convert EPID pixel values into dose values. Some of Varian’s EPID is affected by the backscatter of the support arm, which must be removed before using EPID to measure the dose. Berry et al. [21] and Camilleri et al. [25] established a linear correction relationship for the backscatter of different square fields by comparing the values in the negative inline direction with those in the positive inline or crossline direction. Our study revealed that the dose response of different EPIDs was not the same. Figure 9 shows the ratio of EPID images in the negative inline direction (${G_{\mathrm{EPID}}}^{\left(y\right)}$) and the negative crossline direction (${G_{\mathrm{EPID}}}^{\left(x\right)}$) when the field size was 5, 10, 15, and 20 cm^2^. When the field size was 5 cm^2^, ${G_{\mathrm{EPID}}}^{\left(y\right)}$ < ${G_{\mathrm{EPID}}}^{\left(x\right)}$; when the field size was 10 cm^2^, ${G_{\mathrm{EPID}}}^{\left(y\right)}$ = ${G_{\mathrm{EPID}}}^{\left(x\right)}$; when the field size was 15 and 20 cm^2^, ${G_{\mathrm{EPID}}}^{\left(y\right)}$ > ${G_{\mathrm{EPID}}}^{\left(x\right)}$, and the relationship between the ratio and off-axis distance was closer to the quadratic function relation. Thus, the backscatter correction is not completely linear. In addition, Ko et al. [30] used the MC method to simulate the backscatter kernel of the EPID, but the modeling process is complicated and challenging. In our study, we found that when the field size was ≤10 cm^2^, 10 cm^2^ < field size ≤ 15 cm^2^, and >15 cm^2^, the backscatter effect was different. To remove the effect of EPID backscatter in a simple and accurate manner, we use the measured value of MatriXX as the reference from which to construct the correction matrix. MatriXX is not affected by the support arm’s scatter, so it can be used to accurately correct the EPID backscatter. Therefore, we extracted the corresponding regions of the correction matrices of different fields and combined them into three common correction matrices (Figure 2), which can accurately correct the backscatter of EPID images in different situations. The corresponding correction matrix is selected according to the field size, and there is no need to calculate the backscatter kernel separately for different IMRT or VMAT fields.

The off-axis response of the EPID image changes after dark field and flood field calibration. Renner et al. [28] measured the in-air profile of a 40 × 40 cm^2^ field using the ionization chamber as the off-axis response correction curve, and Camilleri et al. [25] used the ratio of the water dose scanned by the ionization chamber to the EPID pixel value as the off-axis correction matrix. In our model, the backscatter correction matrix was calculated as the ratio of the dose value measured by ICA to the EPID pixel value. The calculation method of this matrix is similar to that of Camilleri et al. [25], so the common correction matrix can also correct the off-axis response of EPID. The response of the corrected EPID image is consistent with the measured value of ICA (Figure 5). Therefore, our model uses the same correction matrix to correct the backscatter and off-axis responses of different EPID images. Due to the low resolution of MatriXX, there may be fewer ionization chambers at the edge of the field, and the measured values may be inaccurate.

We measured the EPID pixel value and the ICA dose value at different off-axis points of the 24 cm^2^ field to obtain the conversion relationship between the pixel and dose values with a larger off-axis distance. We then calculated the conversion coefficient corresponding to different off-axis distances of the corrected EPID image and the uncorrected EPID image. The linear conversion coefficient of each off-axis point in the corrected EPID image is consistent. The same conversion parameters can be used for different off-axis points (central axis) (Table 1 and Table 2). Results show that the dosimetric response of EPID after correction is in accordance with the value measured by the ionization chamber, and the corrected EPID image can truly reflect the output characteristics of the linac. In addition, we established a direct mapping relationship from pixel to dose, which simplified the calculation process without converting the EPID pixel value into fluence and then calculated the dose distribution. For IMRT fields, the gamma pass rates of 3%/3 mm and 2%/2 mm were 99.6% ± 0.94% and 95.48% ± 1.03%, respectively. The EPID calculated dose values were in great agreement with the ICA measured dose values. Therefore, our model could be used to make EPID a clinical dosimetry tool.

## 5. Conclusions

We developed a simple and accurate calibration model for EPID images. A common two-dimensional correction matrix was established to correct the backscatter and off-axis responses of different EPID fields simultaneously. This method does not require use of the MC approach to model the EPID structure or calculate different backscatter correction factors for different fields. Furthermore, this method is suitable for EPIDs with different responses. Our method can use EPID instead of ICA to measure the absolute dose value, rendering the measurement process simpler and more convenient, and the modified EPID can be used for dose verification or to detect patient related errors during radiotherapy.

## Figures and Tables

**Table 1:** Pixel and dose conversion coefficients for different off-axis distances of corrected and uncorrected EPID.

Off-Axis (cm)	EPID-UNCORRECTED		EPID-CORRECTED	
	a	b	a	b
0	0.001398	3.975	0.001398	3.975
1.142	0.001401	3.953	0.001399	3.953
1.904	0.001404	4.148	0.001400	4.148
2.666	0.001406	4.311	0.001401	4.311
3.428	0.001412	4.337	0.001401	4.337
4.190	0.001419	4.424	0.001403	4.424
4.952	0.001423	4.465	0.001402	4.466
5.714	0.001424	4.504	0.001402	4.307
6.476	0.001429	4.633	0.001401	4.432
7.237	0.001435	4.583	0.001402	4.483
7.999	0.001443	4.641	0.001402	4.340
8.761	0.00145	4.701	0.001402	4.300
9.523	0.001462	4.624	0.001403	4.403
10.285	0.001471	4.496	0.001403	4.415
11.047	0.001472	4.496	0.001403	4.423

**Table 2:** The error between the dose value calculated using the central axis conversion coefficient and the measured dose value at different off-axis points.

Off-Axis (cm)	MatriXX (mGy)	EPID-UNCORRECTED (mGy)	Error	EPID-CORRECTED (mGy)	Error
0	1060.77	1056.2	0.4%	1056.2	0.4%
5	1102.98	1078.7	2.2%	1097.2	0.5%
11	1094.20	1035.6	5.4%	1088.9	0.5%

## Data Availability

The data presented in this study are available on request from the corresponding author.
